# An integrated study of hormone-related sarcopenia for modeling and comparative transcriptome in rats

**DOI:** 10.3389/fendo.2023.1073587

**Published:** 2023-02-01

**Authors:** Han Shu, Yubing Huang, Wenqian Zhang, Li Ling, Yuanyuan Hua, Zhengai Xiong

**Affiliations:** ^1^ Department of Obstetrics and Gynecology, The Second Affiliated Hospital of Chongqing Medical University, Chongqing, China; ^2^ Department of Nuclear Medicine, The Second Affiliated Hospital of Chongqing Medical University, Chongqing, China

**Keywords:** hormone, sarcopenia, model, estrogen, RNA-seq, pathogenesis

## Abstract

Sarcopenia is a senile disease with high morbidity, serious complications and limited clinical treatments. Menopause increases the risk of sarcopenia in females, while the exact pathogenesis remains unclear. To systematically investigate the development of hormone-related sarcopenia, we established a model of sarcopenia by ovariectomy and recorded successive characteristic changes. Furthermore, we performed the transcriptome RNA sequencing and bioinformatics analysis on this model to explore the underlying mechanism. In our study, we identified an integrated model combining obesity, osteoporosis and sarcopenia. Functional enrichment analyses showed that most of the significantly enriched pathways were down-regulated and closely correlated with endocrine and metabolism, muscle dysfunction, cognitive impairment and multiple important signaling pathways. We finally selected eight candidate genes to verify their expression levels. These findings confirmed the importance of estrogen in the maintenance of skeletal muscle function and homeostasis, and provided potential targets for further study on hormone-related sarcopenia.

## 1 Introduction

Sarcopenia is a clinical syndrome occurring with aging, characterized by progressive loss of skeletal muscle mass, strength and physical function, always accompanied by weakness, disability and even death. As the average life span increases, the global population aging has been a serious challenge. China is entering a stage of deep aging, with an elderly population aged more than 60 over 267 million and accounting for 18.9% of the total population (1). About 50 million people all over the world are suffering from sarcopenia, and the number is speculated to reach 500 million by 2050 (2, 3). Meanwhile, the menopausal syndrome is an important issue in women’s health care. It is estimated to be 1.2 billion postmenopausal women worldwide in 2030 (4). Hormone insufficiency is one of the risk factors for sarcopenia. Lack of estrogen influences the musculoskeletal system of elderly women and subsequently impairs their physical function and quality of life (5). Taken together, hormone-related sarcopenia is worth more attention.

Accumulated evidence confirmed the importance of sex hormones in muscle homoeostasis (6–8). Estrogen receptors (ER-α and ER-β) are widely expressed in skeletal muscle, suggesting that estrogen has a direct role (8, 9). Through binding to ER-α/β, estrogen may inhibit inflammatory responses and stimulate the activation and proliferation of satellite cells to promote muscle repair (9–13). Besides, estrogen may reduce muscle protein breakdown and/or enhance the sensitivity to anabolic stimuli to maintain muscle mass (13, 14). In addition, estrogen broadly participates in metabolic regulation and mitochondrial function. Estrogen plays a major role in inhibiting mitochondria-dependent apoptosis and ensures efficient energy production in mitochondria probably by suppressing energy dissipation (5, 15–17). Moreover, as an antioxidant and sarcolemmal membrane stabilizer, estrogen could protect the contractility of skeletal muscle from injury (13, 18, 19). Although many effects of estrogen on myoideum are known, the underlying mechanism remains unclear, which is crucial for the prevention and treatment of hormone-related sarcopenia.

Previous studies focused on the metabolic and functional changes of skeletal muscle due to estrogen deficiency, or the effects of estrogen replacement treatment (ERT) on muscle mass and function which still remain in controversy (20–25). Nevertheless, few researches systematically reported the modeling of “hormone-related sarcopenia” from an overall perspective. Additionally, increasing studies have utilized transcriptome technology to explore the pathogenesis of sarcopenia in recent years. Some muscle mass and function related genes such as AMPK, IGF-1, CASK, MGMT, UCP3, strength related genes Tnnc1, TPM3, and other metabolism related genes have been verified (26–30). However, we still lack researches on hormone-related sarcopenia on the transcriptome level.

In our study, we established an ovariectomized (OVX) rat model combined obesity, osteoporosis and sarcopenia. Skeletal muscle fibers are categorized into slow- and fast-twitch fibers (type I and II) (31). In postmenopausal women, the variation of fast-twitch muscle fibers seems to occur preferentially (32, 33). Therefore, we used gastrocnemius (GAS) for subsequent histological and molecular studies. Next, we performed transcriptome sequencing to explore the gene-regulatory circuits associated with hormone-related sarcopenia. Finally, we verified the expression levels of candidate genes from the RNA-Seq data. We hope this study can provide more reference and help to seek potential treatment targets for disease progression in hormone-related sarcopenia.

## 2 Material and methods

### 2.1 Animals

**Figure 1:**
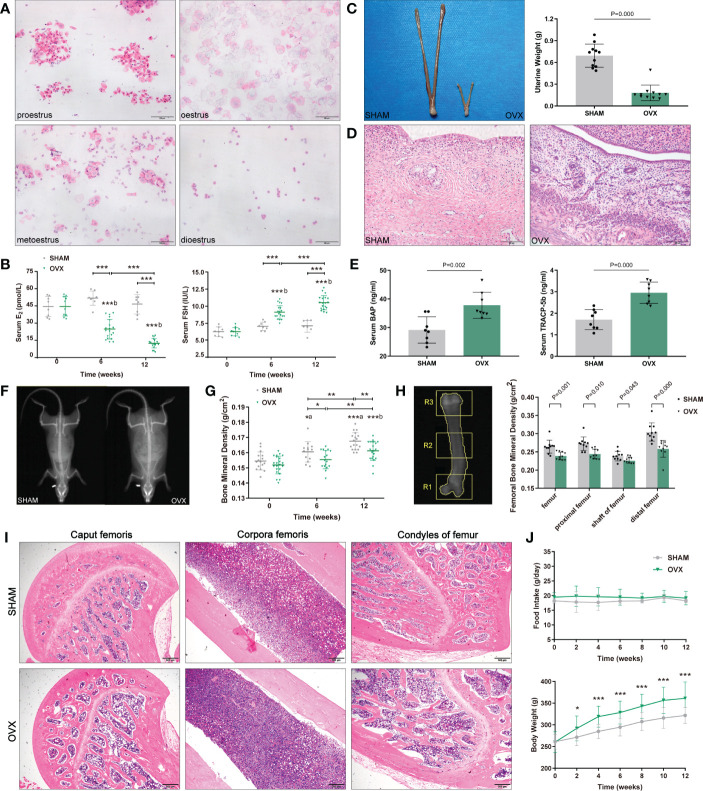
Identification of osteoporosis model in OVX rats. **(A)** 4 sequential stages of vaginal exfoliated cells in regular estrous cycle with H&E. Scale bar, 100μm. **(B)** Serum hormone levels (E_2_, FSH). n≥8 in each group. **(C)** Uterine size and weight. n=11. **(D)** Endometrial and myometrium with H&E. n=11. Scale bar, 100μm. **(E)** Serum bone metabolism markers (BAP, TRACP-5b). n=8. **(F)** The whole-body composition analysis by DXA. **(G)** BMD of the whole-body by DXA. n≥13. **(H)** Femoral BMD by DXA. n=10. R1, R2, R3 corresponded to the proximal, shaft and distal femurs respectively. **(I)** Pathological changes of osteoporosis in femur with H&E. n=5. Scale bar, 500μm. **(J)** Food intake and body weight. n=18. Data are performed as mean ± standard deviation (SD). The statistical significance is expressed as *P<0.05, **P<0.01, ***P<0.001. ‘*a*’ and ‘*b*’ represent the comparison with SHAM-0 and OVX-0 (before modeling) respectively.

Adult female Sprague-Dawley (SD) rats were purchased from the Experimental Animal Center of Chongqing Medical University. Rats were maintained in a pathogen-free facility with a temperature of 22 ± 2°C, a humidity of 60% and a 12/12-hour light/dark cycle. All rats were under barrier conditions with sterile mouse chow and water ad libitum. During the acclimatization weeks, the estrous cycle was recorded by observing vaginal exfoliated cell smears, then the rats with irregular estrous cycle were excluded. A regular estrous cycle includes 4 sequential stages: proestrus, oestrus, metoestrus and dioestrus (**Figure 1A**).

At an age of 12-13 weeks, rats were stratified according to body weight, randomly sampled and underwent bilateral ovariectomy (OVX) or sham surgery (SHAM). Operations were carried out under 2% pentobarbital sodium applied 2mL/kg body weight (BW) intraperitoneally. After shaving and disinfection, the skin of the hypogastrium was incised and the abdomen was opened. In the OVX group, the adnexa were identified, clamped, cut and suture ligated. In the SHAM group, the wound was closed after a similar exposure time without any other procedures. The modeling of hormone-related sarcopenia was evaluated by serum hormones, body weight gain, muscle mass and grip strength (weight-adjusted). At the age of 24-25 weeks (12 weeks after modeling), rats were sacrificed with 3% pentobarbital sodium solution, then the serum, uteri, femurs and GAS were harvested. The GAS samples used for RNA-seq and molecular biological examinations were stored in liquid nitrogen.

This animal study protocol was approved by the Biomedical Ethics Committee of Chongqing Medical University and the Second Affiliated Hospital of Chongqing Medical University prior to performing the study.

### 2.2 Grip strength

To evaluate the physical function of the skeletal muscle, grip strength was tested weekly using an electronic grip strength metre (Cat.47200, Ugo Basil, Italy). After the rats’ forelimbs gripped on the pressure sensor lever, their tails were grasped and pulled back in parallel with the ground until the forepaws released. The maximal reading was recorded every three repetitions.

### 2.3 Body composition analysis

After being tranquilized with 7% chloral hydrate (5mL/kg BW), rats were placed prone below the scanner, and then the body compositions (lean mass, fat and bone mineral density (BMD)) were tested by Dual-energy X-ray absorptiometry (DXA) (Hologic Discovery A, Hologic Inc).

### 2.4 Histological analysis

The uteri, femurs and GAS were fixed, dehydrated, paraffin-embedded and cut into 4-μm-thick sections. The sections were stained with hematoxylin and eosin (H&E). To assess the size of muscle fibers, the average cross-sectional area (CSA) of GAS was analyzed in three randomly chosen areas using Image-Pro Plus software. To distinguish the type of myofibers, some of the GAS were also prepared to be frozen sections for adenosine triphosphatase (ATPase) staining. The count of type I and II myofibers was performed in three randomly chosen fields of 1 mm^2^ in the ATPase stained sections. All the sections were observed under an optical microscope (Olympus Corporation, Japan).

### 2.5 Transmission electron microscopy (TEM)

The isolated fresh GAS tissues were quickly cut into 2 × 2 mm thin slices and fixed in 2.5% glutaraldehyde. After dehydration, infiltration, embedding and polymerization, samples were processed for TEM. The images were captured at ×7k magnification using a transmission electron microscope (HITACHI, Japan). Analysis was performed with Image-Pro Plus software.

### 2.6 Enzyme-linked immunosorbent assay (ELISA)

At 0, 6 and 12 weeks after modeling, serum was obtained from the retro-orbital venous plexus of rats. The FSH and E2, BAP and TRACP-5b levels were quantified using ELISA kits (J&L Biological, China) according to the manufacturer’s instructions.

### 2.7 Immunofluorescence

After deparaffinization, the GAS tissue slides were immersed in EDTA antigen retrieval buffer (pH 8.0) using a microwave, washed with PBS, blocked for 30 minutes in 3% BSA and incubated with anti-ER β antibody (1:200, Aifang Biological, China) overnight at 4°C. The next day slides were washed with PBS and incubated with CY3 goat anti-rabbit lgG antibody (1:150, Aifang Biological, China) for 50 minutes at room temperature. The nuclei were stained with DAPI (Solarbio, China). The scanned images were observed by Slide Viewer software (3DHISTECH, Hungary). The nuclear expression rate of ER-β was measured by Image-Pro Plus software (Media Cybernetics, USA).

### 2.8 RNA-seq and data analysis

The total RNA of GAS tissues was extracted (each group n=5) using TRIzol (Accurate Biology, China) according to the manufacturer’s protocol. The integrity and purity of total RNA were detected using the NanoDrop and Agilent 2100 bioanalyzer (Thermo Fisher Scientific, USA). RNA samples with RIN ≥ 7.0 and 28S/18S ≥ 1.5 were amplified by PCR to construct the qualified cDNA library. The final cDNA library was sequenced using the single-end mode on the MGISEQ-2000 System of the BGI-Tech Bioinformatics Institute (Shenzhen, China).

All profiling analyses for RNA-seq were processed with the assistance of BGI. The raw sequencing data was mapped to the reference genome using the HISAT2 program, aligned to the database built by BGI, and the expression of genes was calculated using the RSEM program. Assessed by principal component analysis (PCA), valid biological replicates in each group were selected for subsequent analysis (n=3). Genes with∣log_2_FC∣≥0.6 and *Q* value<0.05 were identified as the differentially expressed genes (DEGs) utilizing DESeq2. Volcano plot visualization was exerted by ggplot2.

### 2.9 Functional enrichment analysis

Firstly, Gene Ontology (GO) enrichment analysis was executed for three main modules of differentially expressed mRNAs (dif-mRNAs) (*Q* value<0.05). Next, Kyoto Encyclopedia of Genes and Genomes (KEGG) enrichment analysis was performed for pathway sets containing dysregulated mRNAs (*Q* value<0.05) in the molecular biological mechanism network. An enrichment score with a -log10 value (*Q* value) indicates significance for the GO and KEGG pathways terms. Then, the KEGG pathway-act-network was visualized by Cytoscape software (v3.9.1). Moreover, gene set enrichment analysis (GSEA) was conducted for the overall representation of pathways.

### 2.10 Protein-protein interaction (PPI) network construction

The protein-coding genes of dif-mRNAs were submitted to the STRING database for the recognition of possible connections. The minimum interaction score of PPI analysis was set as a high confidence (0.700). After removing disconnected nodes in the network, the data was exported and formed the final visualization of PPI network using Cytoscape software (v3.9.1).

### 2.11 Real-time quantitative polymerase chain reaction (RT-qPCR)

Total RNA of GAS was extracted and the concentration was determined. cDNA was synthesized with the Evo M-MLV RT Mix Kit with gDNA clean (Accurate Biology, China). RT-qPCR was processed with SYBR Green Premix Pro qPCR kit (Accurate Biology, China) using a CFX Real-Time PCR Detection System (Bio-Rad, CA). The equation 2-ΔΔCt was used to calculate the relative fold changes of RNA expression. GAPDH was considered as a reference gene. Relative mRNA expression of the SHAM group was normalized to 1. **Supplementary Table 3** listed all primers of the candidate genes.

### 2.12 Statistical analysis

Statistical analyses were performed and visualized with GraphPad Prism 8.0.1 (San Diego, CA). If failing to pass the Shapiro-Wilk normality test, comparisons of the two groups were made by using the Mann-Whitney nonparametric test. A *P* value <0.05 was regarded as a significant difference. All experiments were repeated at least three times.

## 3 Results

### 3.1 Model-identification of hormone-related Sarco-Osteopenia in SD rats

#### 3.1.1 Identification of menopause

After modeling, the estrous cycles in the OVX group became irregular, and the vaginal exfoliated cells stagnated in dioestrus 3 weeks later. As expected, serum hormones of OVX rats showed a significant decrease in E_2_ and a contrary trend in FSH (**Figure 1B**). Finally, the uteri appeared overall atrophic changes (**Figures 1C, D**).

#### 3.1.2 Identification of osteoporosis

Compared with the SHAM rats, both the serum osteoblast marker BAP and the osteocast marker TRACP-5b of OVX rats increased markedly (**Figure 1E**). The BMD of whole-body in the OVX group were significantly lower than those in the contemporaneous SHAM group (**Figures 1F, G**), and the femoral BMD showed a similar tendency (**Figure 1H**). Besides, the pathological changes in the femur were also in accordance with osteoporosis features: reduction of trabecular bone and expansion of marrow cavity (**Figure 1I**), and the degree of changes in each part accorded with the femoral BMD (the load-bearing parts > the middle part).

#### 3.1.3 Identification of sarcopenia

**Figure 2:**
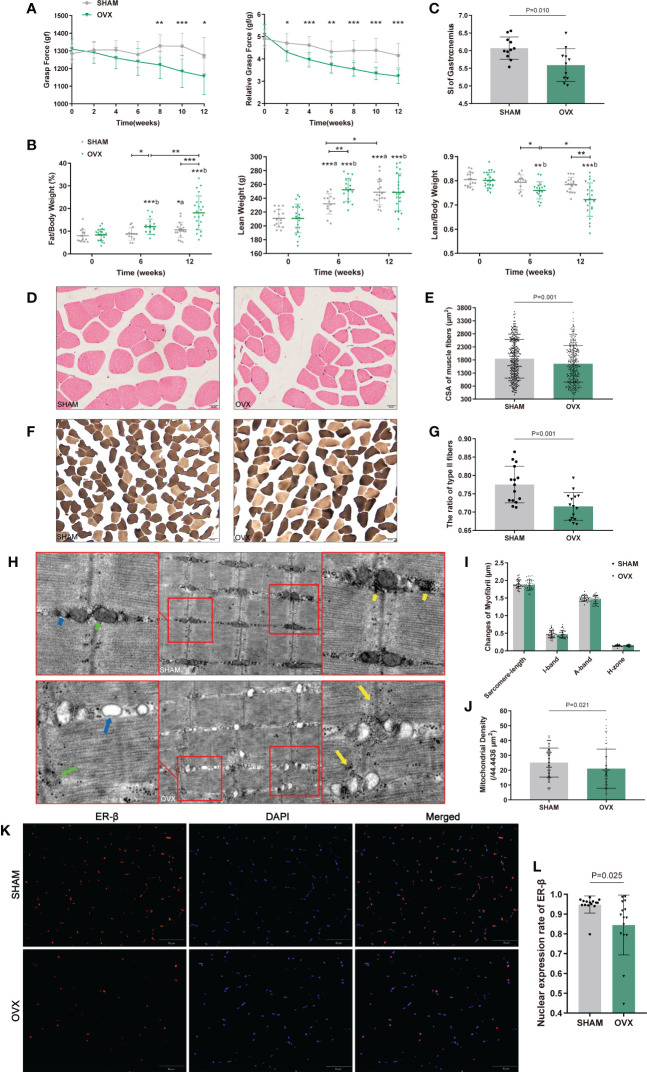
Identification of hormone-related sarcopenia and histological changes in GAS. **(A)** Forelimb grip force and weight-adjusted grip force. n=12 in each group. **(B)** Body compositions analysis by DXA. n≥13. **(C)** Sarcopenia index (SI) of gastrocnemius. n=11. **(D)** Myofibers with H&E staining. n=5. Scale bar, 20μm. **(E)** Quantitative analysis of CSA. n=4 (3 random fields per sample). **(F)** Myofibers with ATPase staining. n=5. Scale bar, 50μm. **(G)** Quantitative analysis of the ratio of type II fibers. n=5 (3 random fields per sample). **(H)** Ultrastructure of myofibrils by TEM. n=6. Scale bar, 1μm. The short green arrow indicates normal mitochondria and the long one indicates abnormal mitochondria; the short blue arrow indicates normal endoplasmic reticulum and the long one indicates swollen endoplasmic reticulum; the short yellow arrows indicate enriched muscular glycogen and the long ones indicate dispersed muscular glycogen. **(I)** Quantitative analysis of myofibrils. n=6 (3 random fields per sample). **(J)** Mitochondrial counts within the unit area. n=6 (10 random fields per sample). **(K, L)** The nuclear expression of ER-β. n=5 (3 random fields per sample). Scale bar, 50μm. Data are performed as mean ± SD. The statistical significance is expressed as *P<0.05, **P<0.01, ***P<0.001. ‘*a*’ and ‘*b*’ represent the comparison with SHAM-0 and OVX-0 (before modeling) respectively.

The body weight of both groups increased with the growth of rats, however, the OVX rats showed a phenotype of severe obesity while the food intake didn’t increase significantly (**Figure 1J**). The forelimb grip force and relative grip force (grip force/BW) were also markedly decreased (**Figure 2A**). As for body compositions (**Figure 2B**), increased body weight was accompanied by increased lean mass in groups, while the final lean mass didn’t differ between groups. Meanwhile, the fat content in the OVX group was greatly higher than that of the SHAM group, indicating that the growth of lean mass in OVX rats was hindered. Correspondingly, the lean mass/BW (relative lean mass) of OVX rats decreased progressively, while that of the SHAM group remained constant. Besides, a significant decrease of sarcopenia index (SI, GAS mass/BW) was also observed in OVX rats (**Figure 2C**). Together, these results of decreased muscle strength and mass (weight-adjusted) demonstrated the success in modeling sarcopenia.

### 3.2 Morphological changes in myofibers

Morphological changes in GAS were observed by H&E, ATPase staining and TEM (**Figures 2D, F, H**). As expected, the CSA and the ratio of type II to total fibers were markedly reduced in the OVX group (**Figures 2E, G**). In the ultrastructure of myofibrils, no significant differences were found in sarcomere-length, I-band, A-band, and H-zone (**Figure 2I**), but the sarcomere damage, swollen endoplasmic reticulum, reduced and dispersed muscular glycogen (**Figure 2F**) and evidently reduced mitochondrion (**Figures 2F, J**) were clearly observed compared with SHAM group. We further detected the variation of ER-β for its more widely distribution than ER-α in skeletal muscle. The ER-β expression in OVX rats also significantly declined (**Figures 2K, L**). These results showed the microscopic histological alterations and ultramicro subcellular structural dysfunction of myofibers with hormone deficiency.

### 3.3 RNA-seq data analysis

#### 3.3.1 Identification of DEGs

**Figure 3:**
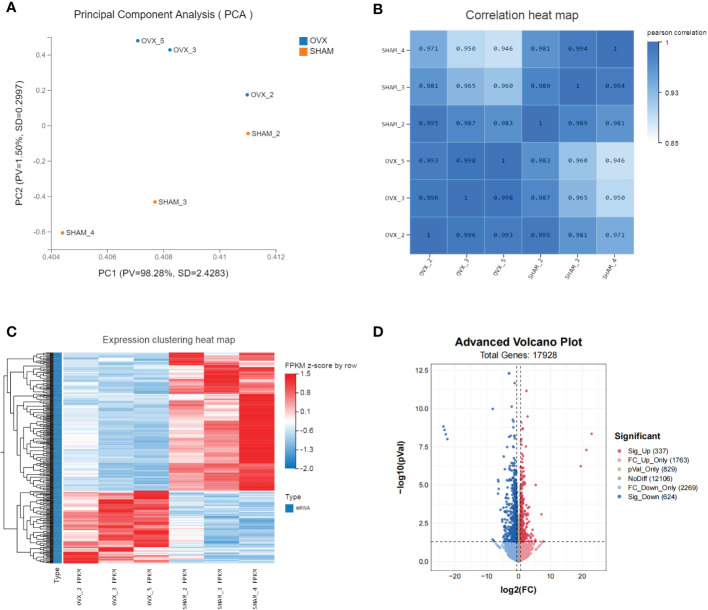
Identification of dif-mRNAs of GAS in SHAM vs. OVX (valid biological replicates n=3 in each group). **(A)** Principal component analysis (PCA). **(B)** Correlation heat map. **(C)** Hierarchical clustering of mRNA sequencing showed mRNA differential expression profiles (|log_2_FC|≥0.6, *Q* value <0.05). Each row indicates a single gene and each column indicates a sample. **(D)** Volcano plot of dif-mRNAs. Red dots represent significantly up-regulated mRNAs with log_2_FC ≥ 0.6, *P* value < 0.05. Pink dots represent up-regulated mRNAs with log_2_FC ≥ 0.6, *P* value ≥ 0.05. Brown dots represent up-regulated mRNAs with -0.6 < log_2_FC < 0.6, *P* value < 0.05. Grey dots represent stable mRNAs. Sky-blue dots represent down-regulated mRNAs with log_2_FC ≤ -0.6, *P* value ≥ 0.05. Blue dots represent significantly down-regulated mRNAs with log_2_FC ≤ -0.6, *P* value < 0.05.

The valid biological replicates in each group were selected (n=3) by PCA and person correlation coefficient (**Figures 3A, B**). A total of 362 DEGs were identified in OVX vs. SHAM. Among them, 125 were up-regulated and 237 were down-regulated. The top 20 dysregulated mRNAs were shown in **Supplementary Table 1**. The heat map and volcano plot showed the expression and distribution of the DEGs (**Figures 3C, D**).

#### 3.3.2 GO and KEGG pathway enrichment analysis

**Figure 4:**
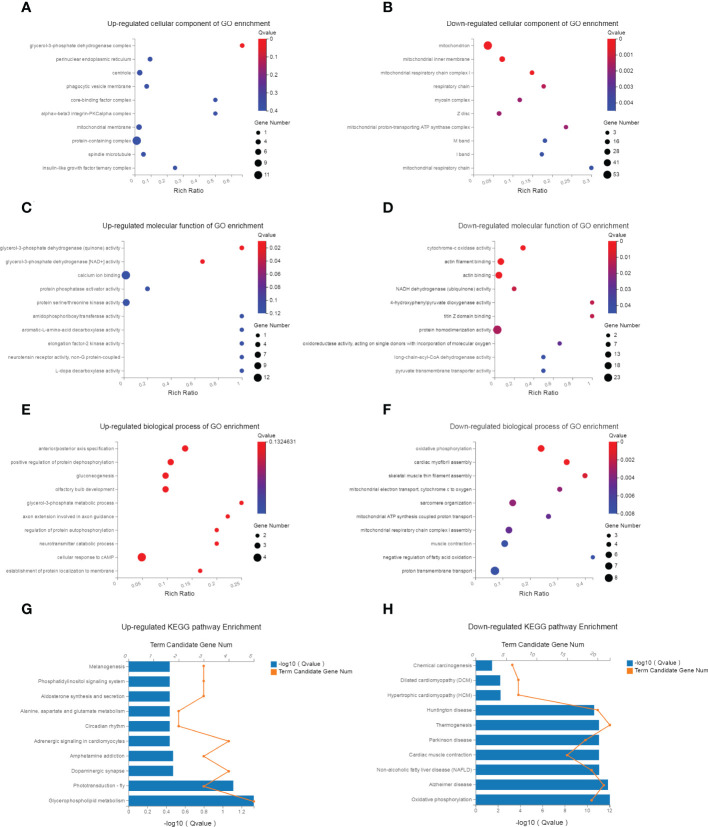
GO and KEGG pathway enrichment analysis. **(A, B)** The top 10 up- and down-regulated KEGG pathway terms of dif-mRNAs. The selection criteria of significant pathways or GO terms was FDR<0.05. **(C, D)** The top 10 up- and down-regulated biological process of GO terms for dif-mRNAs. **(E, F)** The top 10 up- and down-regulated molecular function of GO terms for dif-mRNAs. **(G, H)** The top 10 up- and down-regulated cellular component of GO terms for dif-mRNAs.

GO and KEGG pathway enrichment analyses of the 362 DEGs were performed to identify potential bioinformatic signatures. The enriched up- and down-regulated GO terms in different modules (cellular component, molecular function, biological process) were shown in **Figures 4A–F**, respectively. In the biological process category of GO terms, the top 10 enriched terms were all down-regulated and mainly implicated in oxidative phosphorylation, myofibril assembly, muscle contraction, mitochondrial electron transport and ATP synthesis.

KEGG analysis enriched 187 up-regulated and 206 down-regulated pathways. The up-regulated pathways were significantly related to glycerophospholipid metabolism, adrenergic signaling, circadian rhythm, aldosterone metabolism, phosphatidylinositol signaling system and pathways in cancer (the top 10 were shown in **Figure 4G**). The down-regulated pathways were highly associated with oxidative phosphorylation, Alzheimer disease, muscle contraction and lipid metabolism related diseases, pathways in protein synthesis (the top 10 were shown in **Figure 4H**).

#### 3.3.3 Gene set enrichment analysis (GSEA)

GSEA was next conducted for exhibiting the overall representation of pathways and potential biological mechanisms in diseases. All DEGs in our Seq-RNA data were enriched in 98 up-regulated and 226 down-regulated pathways. The top 50 significant pathways ranked by normalized enrichment score (NES) were shown in **Supplementary Table 2**. Other important pathways were listed in **Supplementary Table 4**. NES>0 means up-regulation while NES<0 means down-regulation. The bigger absolute value of NES suggests the stronger effect of the pathway.

#### 3.3.4 KEGG Pathway-act-network

**Figure 5:**
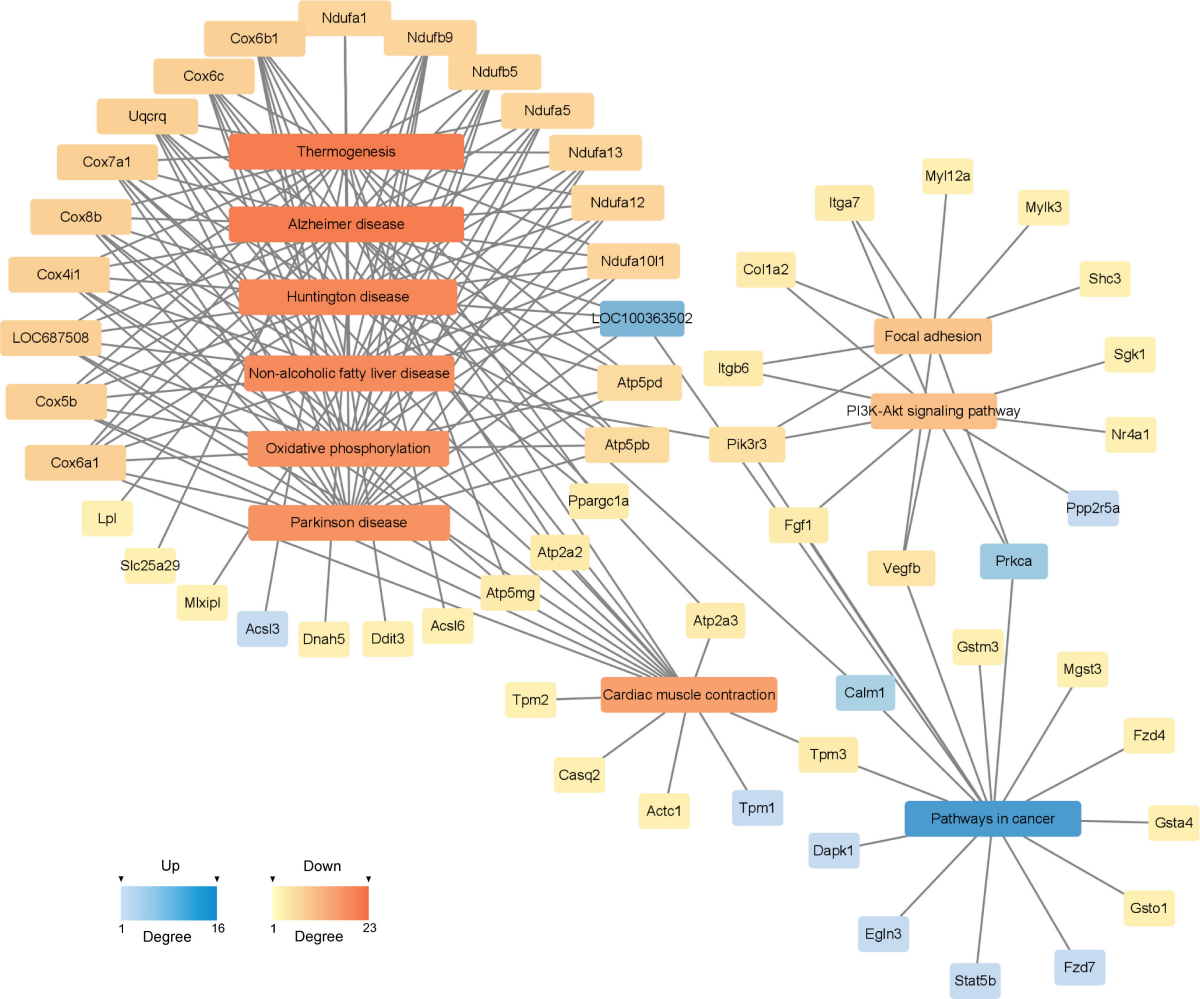
KEGG Pathway-act-network of DEGs. The links and overlaps among dif-mRNAs within top 10 significant enriched KEGG pathway terms. The color and size of each section were both quantified corresponding to the node connections (degree). Blue sections represent up-regulated mRNAs/pathways. Orange sections represent down-regulated mRNAs/pathway.

To investigate the deeper interaction of pathways, 58 dif-mRNAs were enriched into 10 pathways with the most significant interactions, visualized by a constructed KEGG pathway-act-network (**Figure 5**). Furthermore, **Table 1** displays the comparison of the top 10 pathways between KEGG pathway-act-network and GSEA, clearly showing the most important pathways highly associated with the progression of hormone-related sarcopenia. Most of the significantly enriched pathways were down-regulated and implicated in energy metabolism, cardiovascular, neurodegenerative, endocrine and metabolic disease, lipid metabolism.

**Table 1:** The top 10 significant pathways of KEGG pathway-act-network and GSEA.

Num	KEGG pathway-act-network			GSEA		
	KEGG Pathway Term	Node Connections	Regulation	KEGG Pathway Term	Normalized Enrichment Score (NES)	Regulation
1	Thermogenesis	23	down	Parkinson disease	-2.5593	down
2	Alzheimer disease	23	down	Oxidative phosphorylation	-2.5284	down
3	Huntington disease	21	down	Non-alcoholic fatty liver disease	-2.3157	down
4	Non-alcoholic fatty liver disease	20	down	Alzheimer disease	-2.2206	down
5	Oxidative phosphorylation	19	down	Huntington disease	-2.1857	down
6	Parkinson disease	19	down	Cardiac muscle contraction	-2.1626	down
7	Cardiac muscle contraction	16	down	Thermogenesis	-2.1192	down
8	Pathways in cancer	16	up	Ribosome	-1.9247	down
9	PI3K-Akt signaling pathway	10	down	Homologous recombination	1.9221	up
10	Focal adhesion	9	down	Citrate cycle (TCA cycle)	-1.7987	down

The same KEGG pathway terms are maked with red frame.

#### 3.3.5 PPI network construction

**Figure 6:**
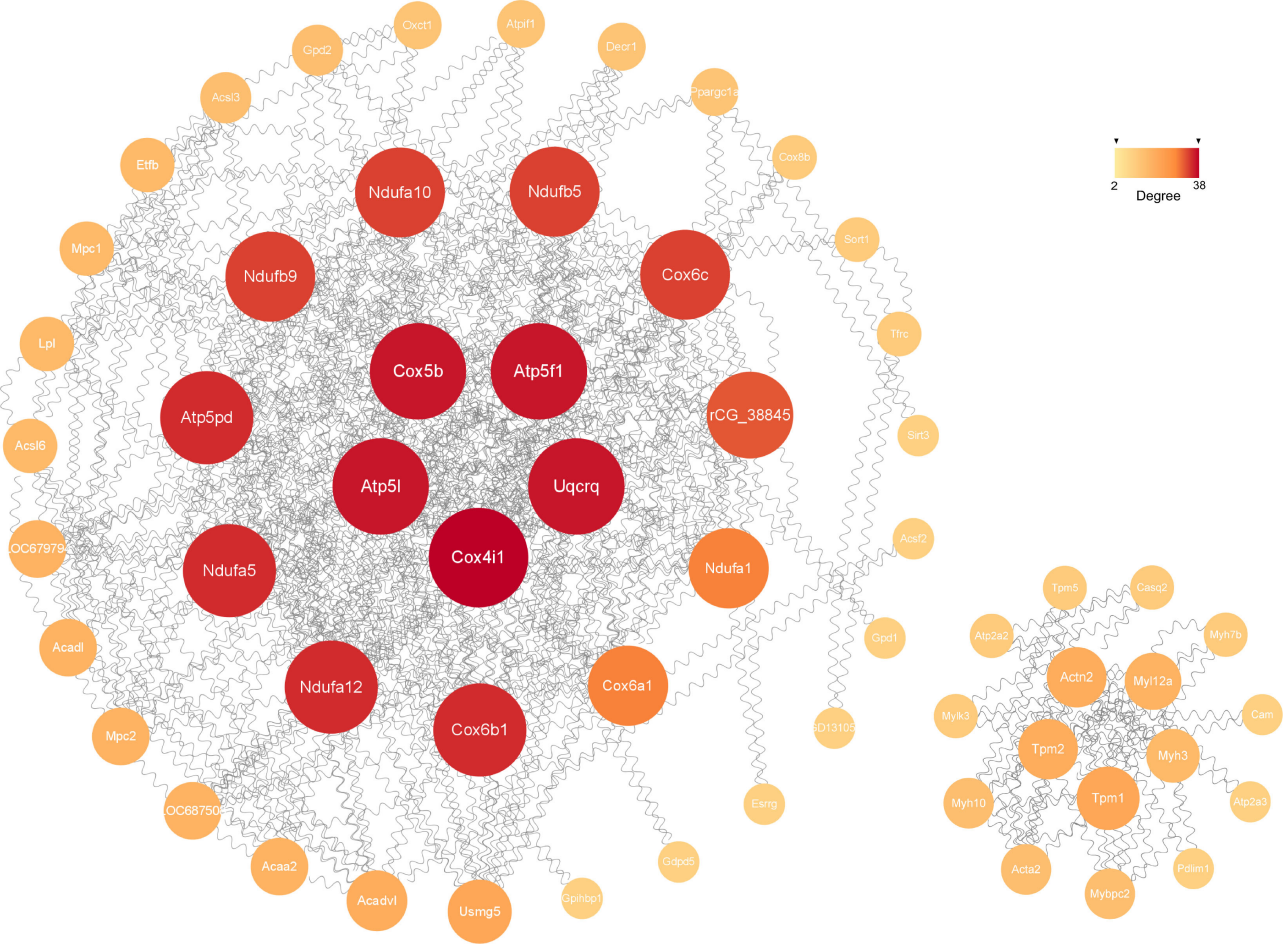
The significant module analysis of PPI network. The color and size of sections were both quantified corresponding to the degree (node connections, range of 2-38).

The possible connections recognized by the STRING consisted of 341 nodes and 240 edges, while the expected edges was only 58, signifying significantly more interactions than expected in the network. As shown in **Figure 6**, the final visualization of 59 key nodes (proteins) were arranged by degree (node connections) in the range of 2-38. The ones closer to the center with darker color and larger size mean a higher degree, playing a more critical role in the PPI.

### 3.4 Validation of the mRNA expression levels of candidate genes by qPCR

**Figure 7:**
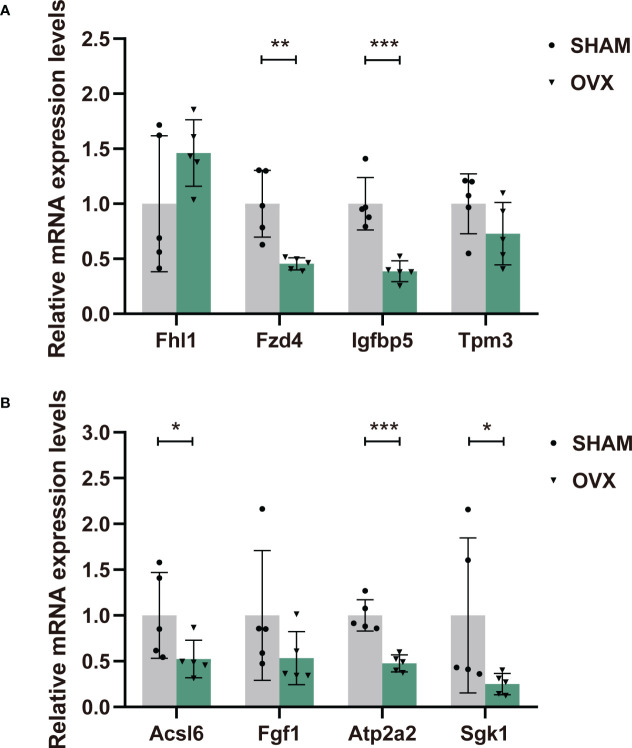
The relative expression levels of dif-mRNAs verified by RT-qPCR. **(A)** Fhl1, Fzd4, Igfbp5, Tpm3. **(B)** Acsl6, Fgf1, Atp2a2, Sgk1. n=5 in each group. Data are performed as mean ± SD. *P < 0.05, **P < 0.01, ***P < 0.001.

Eight candidate genes were selected for qPCR to further confirm the expression levels. They were chosen in consideration of their high expression in both groups, most significant difference in expression between groups (|log2FC|>1) (**Supplementary Table 3**), and high correlations with significant pathways. The expression levels of most genes were consistent with our RNA-seq data except Fhl1 and Igfbp5 (**Figure 7**), which could be owing to the difference in tissues or individuals. In general, RT-qPCR validation of the dysregulated mRNAs in rat models provided us the premise to further study their effects in the hormone-related sarcopenia.

## 4 Discussion

### 4.1 The modeling of hormone-related sarcopenia

In our study, we successfully established a model of sarcopenia by ovariectomy. Compared with the same type of researches, this model has several strengths. Most importantly, we utilized the clinical diagnostic criteria of sarcopenia for modeling identification (2, 34). Firstly, skeletal muscle mass was measured by DXA, which was the preferred method recommended internationally. On this basis, the difference of the muscle mass (weight-adjusted) between groups should not only meet significant statistical difference, but also be greater than twice the standard deviation of the SHAM group (34). Thus, the identification of muscle loss was more accurate and strict. Next, muscle strength tested by grip force (handgrip) was the other indispensable diagnostic element. The revised European consensus on sarcopenia published in 2018 emphasized the low muscle strength as the primary indicator of sarcopenia, and considered it to be the most reliable index for evaluating muscle function (2). When accompanied by a loss in muscle mass, the diagnosis of sarcopenia could be confirmed (2). Besides, this was a sarco-osteopenia model essentially, helping to clarify the alterations and synergetic pathways of musculoskeletal system after menopause. Moreover, monitoring the changes of skeletal system while studying sarcopenia also contributed to verifying our menopausal model, as the ovariectomized model has been widely used to build menopausal osteoporosis. After the definite decline of muscle mass and strength, we systematically described the phenotypic, microscopic and ultramicro changes of muscle tissues.

Skeletal muscle loss is closely related to insulin resistance and metabolic syndromes such as obesity and diabetes (35). As expected, we observed a phenotype of obesity due to estrogen deficiency. In the body composition analysis, the remarkably different body weight and fat mass but similar final lean mass of groups, revealed the essential muscle loss in the OVX rats. Notably, the lean mass grows with the body weight generally, therefore, the evaluation of muscle mass needs to be adjusted by body weight when evident weight difference exists, which means the lean mass/BW is the optimal index for sarcopenia identification (34). With the constant results in the SHAM group as the reference, the relative lean mass in the OVX group decreased steadily. Furthermore, our functional enrichment analyses showed distinct dysfunction in metabolism of energy, lipid, tricarboxylic acid (TCA) cycle, accompanied with increased insulin secretion, providing evidence for the further changes in body compositions caused by estrogen deficiency related metabolic abnormalities.

Not all studies on muscle atrophy were able to detect systemic muscle mass. As the largest and most accessible skeletal muscle in rats and mice, GAS was usually the target tissue. The SI proposed by Edstrom and defined as the target muscle mass/BW (36), was often used as the indicator of sarcopenia. In our study, we calculated SI as an auxiliary reference. Similar to the lean mass, no significant difference was found in GAS mass of groups, and the SI which was weight-adjusted, showed an evidently reduction in OVX rats. Somewhat differently, in studies on age-related sarcopenia, both of the GAS mass and SI declined (37–39). However, the effect of hormone deficiency on sarcopenia is certainly far less than that of aging, which could explain the different levels of muscular atrophy in the two models.

To note, in some studies on an ovariectomized model, no obvious muscle loss was observed (40–42). Inappropriate modeling conditions were probably responsible for this. First, on the premise of relatively weak effect of estrogen deficiency (compared with aging), it demands longer accumulating time after ovariectomy to achieve muscle loss. Another study delivered a similar opinion that reduced estrogen resulted in muscle atrophy in a time-dependent manner (8). Next, the timing of modeling also needs attention. The physical maturity of mice/rats is later than sexual maturity, so the damage of premature operation (earlier than 12 weeks old) on muscle may be concealed by the growth and development of animals. Besides, the selection of target muscle also matters. The alteration owing to estrogen insufficiency in fast-twitch fibers (GAS) often occurs earlier than that in slow-twitch fibers (musculi soleus) (8, 43). Moreover, the sensitivity of rats to hormones makes it more suitable for modeling hormone-related sarcopenia than that of mice (44). After taking all these factors into account, we finally demonstrated the feasibility of modeling the hormone-related sarcopenia by ovariectomy on 12 weeks old rats, with a period of 12 weeks to achieve muscle decline. Based on it, this model can be optimized to choose older but not aged animals or prolong the modeling time, making it more prone to observe typical changes of sarcopenia.

We noticed that the decline in muscle strength was earlier than that in mass. According to the consensus developed by European Working Group on Sarcopenia in Older People (EWGSOP) in 2018, muscle strength was more important than mass in the diagnosis and staging of sarcopenia (2). We also found the high correlation between estrogen and muscle strength in our transcriptome sequencing results (**Figure 4**). Estrogen deficiency gave rise to a markedly down-regulation from myofibril assembly (GO_BP) to muscle contraction (KEGG/GESA), increasing the risk of many diseases with muscle dysfunction.

The size and distribution patterns of muscle fibers changed as expected, and more deeply, the myofibril presented a decline in protein synthesis and energy metabolism after menopause. This ultrastructural injury was also in accord with the top enriched GO_BP terms. These findings confirmed that estrogen insufficiency impaired the structure and function of skeletal muscle, in agreement with similar researches (8, 45, 46). Moreover, decreased expression of ER-β showed evidence for the impaired estrogen-ER signaling, and we subsequently obtained the corresponding results of down-regulated expression of ESRRG (Estrogen-related Receptor Gamma) (log2FC -1.046)) and the estrogen signaling pathway, which possibly accounted for the consequent histologic findings of myofibers. As known, estrogen mediates its cellular actions through nuclear- and membrane-initiated steroid signaling. The “nuclear” way is the classical mechanism mediated by nuclear receptors ER-α and ER-β. Upon activation of ER, various signaling pathways (i.e. PI3K-Akt, Ca^2+^, cAMP, protein kinase cascades) are activated and ultimately influence downstream transcription factors (47).

### 4.2 Potential pathogenesis of hormone-related sarcopenia

The hormone-related sarcopenia shares similar characters with the age-related sarcopenia. Nevertheless, postmenopausal women take more risks of musculoskeletal disorders than men of the same age (46–48). Current clinical treatments for sarcopenia are limited to exercise and diet intervention and quite deficient in medicine (34, 49, 50). The concrete mechanisms of progression in hormone-related sarcopenia still remain obscure.

RNA-seq technology was applied to explore the underlying mechanisms. Some of the results have been stated above. Our various enrichment analyses concentrated in pathways of energy metabolism, lipid metabolism, cardiovascular, neurodegenerative, endocrine and metabolic disease, all of which were highly coincident with the clinically proven long-term risks of menopause (51–55). Furthermore, as shown in **Supplementary Tables 2, 4**, a cascade of signal transduction pathways such as Ras, PI3K-Akt, FoxO, AMPK, MAPK, VEGF, Jak-STAT, TNF, NF-kappa B were down-regulated. These pathways are extensively involved in regulation of cell proliferation, survival, growth, migration, differentiation or cytoskeletal, as well as immunity, inflammation and oxidative stress. Other down-regulated signal transduction pathways like phosphatidylinositol, cAMP, cGMP-PKG and Calcium pathway participate in physiological processes of cell secretion, metabolism, calcium homeostasis and muscle contraction. We also found a down-regulation in HIF-1 signaling pathway. HIF-1 functions as a master regulator of oxygen homeostasis, including oxidative stress, damage, angiogenesis, vascular remodeling, inflammatory reaction, and metabolic remodeling (56). However, how this pathway acts in skeletal muscle requires further study.

The PI3K/Akt pathway was early proved involved in the anti-apoptotic effects of estrogen mediated by ER-α and ER-β in skeletal muscle cells, especially at the mitochondrial level (57, 58). FoxO transcription factors are strongly implicated to multiple regulatory and signaling pathways of oxidative phosphorylation, inflammatory signaling, TCA cycle, and mitochondrial function (59). It was found that muscle atrophy could be ameliorated by regulating myostatin-mediated PI3K/Akt/FoxO3a pathway and satellite cell function in chronic kidney disease (60). Besides, the inhibition of Glut4/AMPK/FoxO pathway could down-regulate the expression of Atrogin1 and MuRF1, and inhibit skeletal muscle protein degradation (61). VEGF signaling pathway would be disrupted when the estrogen-ER signaling was down-regulated, ultimately developiong the coronary microvasculature in the heart (62). The Jak/STAT pathway is required for the homeostasis of different tissues and organs, and when deregulated, it promotes the initiation and progression of pathological conditions including cancer, obesity, diabetes and other metabolic diseases (63, 64). Under an inflammatory environment, various pro-inflammatory cytokines directly act on Jak/STAT, p38MAPK and NF-κB pathways and then participate in muscle atrophy (65, 66).

Among the up-regulated pathways, we are also attentive to the ubiquitin-mediated proteolysis, steroid biosynthesis, GnRH, TGF-beta and IL-17 signaling pathway (**Supplementary Table 2**). The change of steroid biosynthesis and GnRH signaling pathway was in response to the endocrine regulation by hypothalamo-pituitary-gonadal axis. Besides, the maintenance of skeletal muscle mass depends on an overall balance between the protein synthesis and degradation. Growing evidence implicates the ubiquitin-mediated proteolysis is a key contributor to muscle loss (67). As for the IL-17 family, it plays a crucial role in both acute and chronic inflammatory responses (68).

Currently, there is a growing number of researches on transcriptome for sarcopenia, and most of them are regarding age-related sarcopenia. Age-related muscular changes of gene expression in healthy individuals often reflect accumulated damage and compensatory adaptations for preservation of tissue integrity. To characterize these changes, an RNA-seq study was performed from muscle biopsies of 53 healthy individuals aged 22-83 years old (69). The most differentially abundant mRNAs encoded proteins implicated in age-related processes of cellular senescence, insulin signaling and myogenesis. Then specific mRNA isoforms that changed significantly with aging were enriched for proteins involved in oxidative phosphorylation and adipogenesis. Moreover, alternative splicing of some genes were found changing systematically with aging, and tended to cluster in proteins involved in oxidative phosphorylation, lipid metabolism and MTORC1 regulation, which might be compensatory to the decline of mitochondrial function with aging. In previous studies, the mRNAs such as FAM83B, C12orf75, LPP, SKAP2, CRIM1, FEZ2, LGI1 and MYLK4 have already been linked to muscle aging (70–74). Members of FAM protein family (FAM151a, FAM13a, FAM131a, FAM20c), LGI1 and MYLK3 were also significant DEGs in our study (|log_2_FC|>1). Another transcriptome study analyzed the gene expression data files of human and rats from the Gene Expression Omnibus (GEO) database (27). Results showed that the expression of mitochondria genes involved in mitochondrial electron transport, complex assembly of the respiratory chain, TCA cycle, oxidative phosphorylation and ATP synthesis were down-regulated in skeletal muscle with aging. Then a primary hub gene of CYCS (Cytochrome C) and a key transcription factor of ESRRA (Estrogen-related Receptor Alpha) were further identified to be associated closely with skeletal muscle aging. Accordingly, the main cause of skeletal muscle aging may be the systematically reduced expression of mitochondrial functional genes. Similarly, we observed a markedly down-regulation of ESRRG. However, the knowledge of the functions of these genes is limited, especially in the skeletal muscle and aging.

There were very few transcriptome studies of hormone-related sarcopenia. A previous study on estrogenic regulation of skeletal muscle in pre- and post-menopausal women, suggested for the first time at human proteome level, that estrogen was a major regulator of human skeletal muscle signaling in women (75). The major canonical pathways which were differentially regulated included mitochondrial dysfunction, oxidative phosphorylation, glycolysis and TCA-cycle, strong indicators for affected energy metabolism. The major biological processes predicted to be affected were related to cell death, apoptosis and cell survival, as well as contractility of the muscle and glycolysis. Furthermore, estrogen was confirmed to be an upstream regulator of these processes *in vitro*. An earlier transcriptome study first reported the changes in the skeletal muscle of postmenopausal women (24). Integrated results revealed transcription level changes in, e.g., muscle protein and energy metabolism, indicating that menopause with estrogen deprivation might accelerate the deterioration in aging muscles. In particular, the ubiquitine-proteosome system was effected at several levels. Hormone replacement therapy (HRT) seemed to partially counteract the hormone-related transcriptional changes and therefore aid in maintaining proper muscle mass and function after menopause. Nevertheless, the mRNA levels of ESR1 and ESR2 were differentially expressed but not regulated by HRT.

Interestingly, we also found commonalities between the hormone-related and diabetes-related sarcopenia. According to a latest research, the diabetes-induced sarcopenia model of db/db mice shared a similar phenotype of obesity, osteoporosis and sarcopenia with the OVX rats (28). Correspondingly, functional enrichment analysis of this model represented significantly down-regulated pathways of oxidative phosphorylation, metabolic pathways and muscle contraction. Considering the importance of estrogen in the endocrine regulation, these commonalities with diabetes-induced sarcopenia were likely attributable to the extensive effects on systemic energy metabolism by estrogen.

The construction of PPI network is also conducive for mining core regulatory genes (**Figure 6**). The series of encoded proteins (Ndufa12, Ndufa5, Ndufb9, etc) are subunits of the NADH: ubiquinone oxidoreductase (complex I) which is part of the oxidative phosphorylation system in mitochondria, participate in oxidative phosphorylation, thermogenesis, Non-alcoholic fatty liver disease (NAFLD) and diseases of Alzheimer, Parkinson, Huntington. The Uqcrq (part of the ubiquinol-cytochrome C reductase complex III) and cytochrome C oxidase subunits (Cox4i1, Cox5b, Cox6b1, etc), as well as the ATP synthase subunits (Atp5l, Atp5f1, Atp5pd, etc), also take part in the biological processes above. Moreover, the cytochrome C oxidase and reductase are important participants in muscle contraction. Meanwhile, the Actn2 (actinin alpha 2), tropomyosin (Tpm1, Tpm2), and constituents of myosin (Myl12a, Myh3, Myh10, etc) provide driving force for muscle contraction. Besides, the proteins of Atp2a2 and Atp2a3 are responsible for Ca^2+^ transporting in the sarcoplasmic/endoplasmic reticulum of ATPase, affecting muscle contraction through the Calcium, cGMP-PKG and cAMP signaling pathway. Additionally, The acyl-CoA dehydrogenase and transferase (Acadvl, Acadl, Acaa2) are involved in the process of fatty acid metabolism.

With comprehensive analyses of the transcriptome sequencing, we chose eight candidate genes for further validation of their expression levels. There were several filtering conditions for selection. Firstly, the greater the inter-group difference in gene expression, the higher the correlation with our intervention, the lower the possibility of false positive results. Secondly, the gene with high expression in both groups was easier to be detected. Thirdly, it really mattered weather it was an upstream gene or core gene of the pathway we paid attention to, with the reference of the KEGG Pathway Map and GSEA.

Our functional enrichment analyses have given prominence to the most important pathways involved in the development of hormone-related sarcopenia. The selected genes extensively participate in multiple pathways. According to GESA, Acsl6 was a core gene in pathway “fatty acid degradation/metabolism/biosynthesis”, “PPAR signaling”, “peroxisome” and “thermogenesis”. Evidence from models implicates long-chain acyl-CoA synthetases (ACSLs) as key regulators of skeletal muscle fat oxidation and storage, and suggests distinct roles for different Acsl6 isoforms in skeletal muscle fat metabolism (76–78). In a study aimed to determine the relationship between Acsl6 and fat oxidation and storage, compared with low-fat diet (LFD), the high-fat diet (HFD) and exercise training both resulted in greater Acsl6 protein abundance, while under fasted condition, skeletal muscle Acsl6 protein abundance was positively correlated with intramyocellular lipid content (76). As a potential regulator of glucose homeostasis, Fgf1 could exert superior glycemic control by targeting skeletal muscle (79). Besides, a study of myogenic cell differentiation in skeletal muscle derived stem cells showed, with a robust myogenic effect of the satellite cells, the expression of Fgf1 increased (80), which could explain our result. Also, Fgf1 was a core gene in pathway “regulation of actin cytoskeleton”, “Rap1 signaling” and “Ras signaling”. Fgf1 and Sgk1 were both the core genes in PI3K-Akt signaling pathway. Sgk1 is also closely related to regulation of the Foxo and mTOR signaling pathway. It has been reported that Sgk1 is critical for maintaining skeletal muscle homeostasis and function. The activation of Sgk1 might down-regulate proteolysis and autophagy as well as increase protein synthesis (81, 82), providing support for our result.

Next, the Tpm3 and Atp2a2 were core genes in pathway “cardiac muscle contraction”, “dilated cardiomyopathy” and “hypertrophic cardiomyopathy”. As genes that directly affect muscle contraction, their expression in muscle dysfunction diseases always decline (83–86), which was also validated in out result. Besides, Atp2a2 played a crucial role in pathway of “Alzheimer disease”. Fhl1 is involved in Jak-STAT signaling pathway, and highly associated with the illness of myatrophy. Mutation of Fhl1 is the causative factor of several X-linked hereditary myopathies that are collectively termed Fhl1-related myopathies. These disorders are characterized by severe muscle dysfunction and damage. One study found that, loss of Fhl1 induced an age-dependent skeletal muscle myopathy associated with myofibrillar and intermyofibrillar disorganization in mice (87). However, another study suggested that the expression of Fhl1 might activate myostatin signaling in skeletal muscle and promote atrophy under the appropriate condition (88). The validation of Fhl1 expression in the OVX group was increased, contrary to the sequencing result. Nevertheless, the correct expression trend has not been defined yet, and more verification is needed. Additionally, Fzd4 is an important upstream molecule of Wnt signaling pathway. Fzd4 was found to increase cardiomyocyte output through the canonical Wnt pathway (89). It was also differentially expressed in a skeletal muscle transcriptome study of postmenopausal women (90). Finally, the Insulin-Like Growth Factor (IGF) family play an important role in regulating cell proliferation, differentiation, apoptosis and transformation. As a member of the IGFs, Igfbp5 partakes in regulation of cell growth and fibronectin binding. A vitro experiment verified that the decreased level of Igfbp5 protein restrained the proliferation and differentiation of bovine skeletal muscle satellite cells (91). Conversely, in a recent study, increased expression of Igfbp5 was detected in skeletal muscle biopsies of spinal muscular atrophy (SMA) patients and non-SMA neuromuscular diseases (92). In our study, the decreased expression of Igfbp5 in qPCR validation was also inconsistent with the transcriptome sequencing, which was probably attributed to the difference in muscle tissues or individuals. In general, our validation of the eight genes accorded with the Seq-RNA, and the results could be supported or explained by other relevant studies. However, to clarify the more specific mechanisms, further work on protein levels will also be necessary.

In conclusion, we systematically studied the development of hormone-related sarcopenia combined with bioinformatics analysis. This model is applicable for studying the effect of sex hormones on sarcopenia without the interference of aging. The phenotype and pathogenesis of sarcopenia caused by different inducements (aging, HFD and hormone deficiency) have similarities. Owing to the unique sexual characteristics, however, the hormone-related sarcopenia owns its particularity. Our findings supported the viewpoint that muscle protein synthesis, autophagy and ubiquitin-mediated proteolysis are key contributors to muscle loss. Apart from the classical hormone-related pathways, we also identified many pathways closely correlated with endocrine and metabolism, muscle dysfunction and cognitive impairment. Furthermore, the validated genes and some signaling pathways rarely reported in researches on sarcopenia, for example, VEGF, cGMP-PKG and HIF-1 signaling pathway, are potential to be therapeutic targets.

## Data availability statement

The data presented in the study are deposited in the NCBI-SRA repository, accession number PRJNA892241

## Ethics statement

The animal study was reviewed and approved by the Biomedical Ethics Committee of Chongqing Medical University and the Second Affiliated Hospital of Chongqing Medical University.

## Author contributions

HS conducted animal experiments, performed bioinformatic analyses and wrote this manuscript. YubH assisted in animal experiments and bioinformatic analyses. WZ and LL provided technical guidance for experiments. YuaH and ZX provided ideas for this study, and ZX revised this manuscript. All authors contributed to the article and approved the submitted version.
